# *Staphylococcus aureus* Cell Wall Biosynthesis Modulates Bone Invasion and Osteomyelitis Pathogenesis

**DOI:** 10.3389/fmicb.2021.723498

**Published:** 2021-08-16

**Authors:** Elysia A. Masters, Gowrishankar Muthukrishnan, Lananh Ho, Ann Lindley Gill, Karen L. de Mesy Bentley, Chad A. Galloway, James L. McGrath, Hani A. Awad, Steven R. Gill, Edward M. Schwarz

**Affiliations:** ^1^Center for Musculoskeletal Research, University of Rochester Medical Center, Rochester, NY, United States; ^2^Department of Biomedical Engineering, University of Rochester Medical Center, Rochester, NY, United States; ^3^Department of Orthopaedics, University of Rochester Medical Center, Rochester, NY, United States; ^4^Department of Microbiology and Immunology, University of Rochester Medical Center, Rochester, NY, United States; ^5^Department of Pathology and Laboratory Medicine, University of Rochester Medical Center, Rochester, NY, United States

**Keywords:** *S. aureus*, osteomyelitis, cell wall, PBP 3, autolysin, surface adhesion, osteocyte canaliculi, osteolysis

## Abstract

*Staphylococcus aureus* invasion of the osteocyte lacuno-canalicular network (OLCN) is a novel mechanism of bacterial persistence and immune evasion in chronic osteomyelitis. Previous work highlighted *S. aureus* cell wall transpeptidase, penicillin binding protein 4 (PBP4), and surface adhesin, *S. aureus* surface protein C (SasC), as critical factors for bacterial deformation and propagation through nanopores *in vitro*, representative of the confined canaliculi *in vivo*. Given these findings, we hypothesized that cell wall synthesis machinery and surface adhesins enable durotaxis- and haptotaxis-guided invasion of the OLCN, respectively. Here, we investigated select *S. aureus* cell wall synthesis mutants (Δpbp3, Δatl, and ΔmreC) and surface adhesin mutants (ΔclfA and ΔsasC) for nanopore propagation *in vitro* and osteomyelitis pathogenesis *in vivo*. *In vitro* evaluation in the microfluidic silicon membrane-canalicular array (μSiM-CA) showed *pbp3*, *atl*, *clfA*, and *sasC* deletion reduced nanopore propagation. Using a murine model for implant-associated osteomyelitis, *S. aureus* cell wall synthesis proteins were found to be key modulators of *S. aureus* osteomyelitis pathogenesis, while surface adhesins had minimal effects. Specifically, deletion of *pbp3* and *atl* decreased septic implant loosening and *S. aureus* abscess formation in the medullary cavity, while deletion of surface adhesins showed no significant differences. Further, peri-implant osteolysis, osteoclast activity, and receptor activator of nuclear factor kappa-B ligand (RANKL) production were decreased following *pbp3* deletion. Most notably, transmission electron microscopy (TEM) imaging of infected bone showed that *pbp3* was the only gene herein associated with decreased submicron invasion of canaliculi *in vivo*. Together, these results demonstrate that *S. aureus* cell wall synthesis enzymes are critical for OLCN invasion and osteomyelitis pathogenesis *in vivo*.

## Introduction

*Staphylococcus aureus* is a ubiquitous organism of the human microbiota colonizing the nares of approximately 30% of individuals (Kluytmans et al., 1997). Despite its ability to asymptomatically colonize a large percentage of the population, *S. aureus* can also cause severe disease as an opportunistic pathogen (Lowy, 1998). In the setting of implant-associated bone infection, *S. aureus* has evolved to express various virulence mechanisms that enhance its survival and ability to evade host immunity.

While many pathogens have been reported to cause prosthetic joint infections (Parvizi et al., 2008), clinically *S. aureus* remains the most important infectious pathogen to date. It is not only the most prevalent pathogen in implant-associated osteomyelitis (Arciola et al., 2005; Pulido et al., 2008) but also the most destructive. Moreover, *S. aureus* infection of the bone is considered very difficult to cure (Tomizawa et al., 2020; Masters et al., 2021), due to specific mechanisms that enable bacterial survival within the implant and bone microenvironment following revision surgery and antibiotic therapy [reviewed in Masters et al. (2019b) and Muthukrishnan et al. (2019)].

Our discovery of *S. aureus* invasion of the osteocyte lacuno-canalicular network (OLCN) of the cortical bone, initially described in murine models for implant-associated osteomyelitis (de Mesy Bentley et al., 2017) and later validated in human diabetic foot infections (de Mesy Bentley et al., 2018), has become a prominent area of active research. In order to invade the submicron-sized canaliculi of the OLCN, *S. aureus* must deform from a 1-μm cocci to an elongated “rod-shaped” cell, measuring as small as 0.2 μm in diameter (de Mesy Bentley et al., 2017). This submicron-scale invasion of bone permits *S. aureus* long-term survival and evasion of immune cell attack. Despite the challenges associated with identifying *S. aureus* bacterial cells deep within the infected bone, like finding a needle in a haystack, additional studies in models of fracture-related infection and implant-associated infection have been able to corroborate this novel mode of persistence (Alder et al., 2020; Zoller et al., 2020).

Toward elucidation of druggable targets to effectively treat chronic osteomyelitis, recent studies aimed to determine the genetic mechanism of *S. aureus* deformation and propagation through the OLCN. Previous work developed an *in vitro* model called the microfluidic silicon membrane-canalicular array (μSiM-CA) to mimic the physiologic dimensions of canaliculi to screen a library of *S. aureus* transposon insertion mutants (Masters et al., 2019a, 2020). These studies showed that deletion of *pbp4*, encoding the cell wall transpeptidase penicillin binding protein 4 (PBP4), significantly reduced *S. aureus* propagation through nanopores *in vitro* and eliminated *S. aureus* invasion of the OLCN while also decreasing the extent of pathogenic bone loss at the infection site in a murine model of implant-associated osteomyelitis (Masters et al., 2020). However, the mechanism of PBP4 involvement in OLCN invasion, as well as modulation of pathogenic bone loss, remains unclear. In addition, expression of *S. aureus* surface protein C (SasC) was critical for *S. aureus* deformation and propagation through nanopores (Masters et al., 2020) *in vitro* but has not yet been validated for OLCN *in vivo*.

**FIGURE 1:**
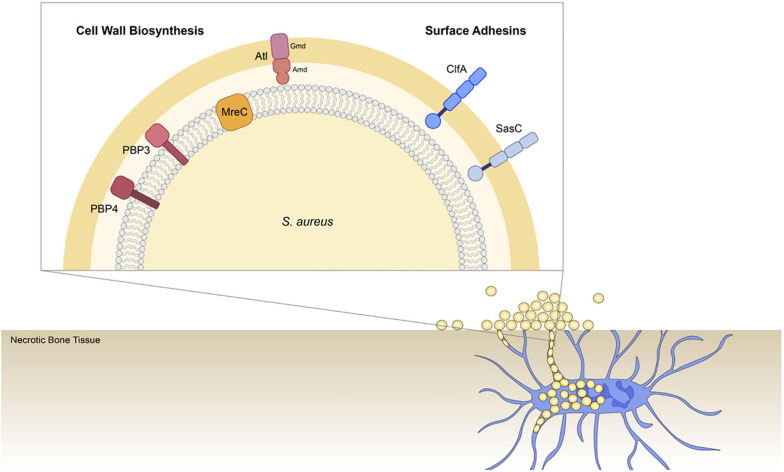
Illustration of *Staphylococcus aureus* genes investigated for their role in osteocyte lacuno-canalicular network (OLCN) invasion during osteomyelitis. To expand our understanding of *S. aureus* deep bone infection, this work investigated selected cell wall biosynthesis proteins and surface adhesins. Previous studies identified PBP4 as a critical factor of *S. aureus* OLCN invasion *in vivo* (Masters et al., 2020). Here we investigated the non-essential transpeptidase, penicillin binding protein 3 (PBP3; Pinho et al., 2000); proposed morphogenetic determinant protein, cell shape-determining protein MreC (MreC) (Divakaruni et al., 2007); major peptidoglycan hydrolase, Autolysin (Atl), which is composed of Amd and Gmd subunits (Vollmer et al., 2008); cell wall-anchored and fibrin binding protein, clumping factor A (ClfA) (McDevitt et al., 1994); and cell wall-anchored protein involved in biofilm formation, surface protein C (SasC; Zhu et al., 2020).

We hypothesize that *S. aureus* invades the OLCN via the guidance of durotaxis and haptotaxis cues, which are defined as motility guided by substrate stiffness (Sunyer et al., 2016; Sunyer and Trepat, 2020) and three-dimensional (3D) extracellular matrix (ECM) organization (Hsu et al., 2005; Oudin et al., 2016), respectively. Therefore, the current study aims to build upon previous work by investigating the role of select *S. aureus* genes across different functional groups in osteomyelitis pathogenesis to improve our understanding of *S. aureus* OLCN invasion. Specifically, this work investigates cell wall biosynthesis proteins: penicillin binding protein 3 (PBP3), autolysin (Atl), and cell shape-determining protein MreC (MreC), hypothesized to mediated durotaxis; and surface adhesin proteins: clumping factor A (ClfA) and SasC, hypothesized to mediate haptotaxis (Figure 1).

The bacterial cell wall is primarily composed of peptidoglycan, whose synthesis is catalyzed by PBPs. *S. aureus* has four genome-encoded PBPs (1–4), where PBP1 and PBP2 are essential proteins for cell wall synthesis (Sauvage et al., 2008). PBP3 and PBP4 are both non-essential, monofunctional PBPs with only transpeptidase activity (Pinho et al., 2000; Scheffers and Pinho, 2005). PBP4 is the only low-molecular-weight PBP of *S. aureus* and is largely responsible for high degrees of muropeptide cross-linking in the cell wall (Navratna et al., 2010). PBP3 is considered a class B, high-molecular-weight PBP, and its role in the *S. aureus* cell cycle is largely unknown (Yoshida et al., 2012; Kylväjä et al., 2016). Balancing the activity of the PBPs are *S. aureus* autolysins, or peptidoglycan hydrolases, which degrade the cell wall during growth and division. Atl is the major peptidoglycan hydrolase of *S. aureus* and is composed of amidase (Amd) and glucosaminidase (Gmd) subunits (Vollmer et al., 2008). In addition to its role in cell wall hydrolysis during cell division (Varrone et al., 2011, 2014), Atl also has the ability to bind ECM ligands as a surface adhesin and therefore may be involved in both haptotaxis and durotaxis functions (Clarke and Foster, 2006; Bose et al., 2012; Hirschhausen et al., 2012; Schlesier et al., 2020). Lastly, MreC is a morphogenetic determinant protein in rod-shaped bacteria (Carballido-López and Formstone, 2007; Divakaruni et al., 2007) but is conserved in *S. aureus* without a known function (Tavares et al., 2015). Previous studies showed that a *mreC* mutant trended toward decreased ability to propagate through nanopores *in vitro*, suggesting a possible role in OLCN invasion (Masters et al., 2020).

In addition to cell wall synthesis machinery, *S. aureus* surface adhesins were investigated for their role in OLCN invasion. *S. aureus* has a broad range of cell wall-anchored surface adhesin proteins, which are important for bacterial virulence and survival (Foster et al., 2014). Here, we investigate surface adhesins ClfA and SasC. ClfA is a cell wall-anchored protein that binds fibrin(ogen) (McDevitt et al., 1994), involved in abscess formation (Cheng et al., 2011) and infection severity in diabetic murine models (Farnsworth et al., 2017). SasC is a cell wall-anchored protein involved in cell aggregation and biofilm accumulation (Schroeder et al., 2009; Zhu et al., 2020). As mentioned, SasC was identified in previous studies as a statistically significant gene for nanopore propagation but was not validated with gene deletion and evaluated *in vivo* (Masters et al., 2020).

This study applied *in vitro* and *in vivo* methods to investigate the role of *S. aureus* cell wall synthesis machinery and surface adhesins in OLCN invasion. Here, we observe similarities between infection phenotypes of *S. aureus pbp4* and *pbp3* deletion mutants, including hindered OLCN invasion. Deletion of cell wall synthesis genes *pbp4*, *pbp3*, and *atl* showed modulated infection pathogenesis with altered abscess formation and decreased pathogenic bone loss, while *mreC* expression did not have a role in *S. aureus* implant-associated osteomyelitis. In contrast, deletion of surface adhesin genes *clfA* and *sasC* had marginal effects on infection pathogenesis in implant-associated osteomyelitis, leading to the conclusion that durotaxis may be the primary mechanism for *S. aureus* OLCN invasion and propagation.

## Materials and Methods

### Strains and Growth Conditions

*Staphylococcus aureus* USA300 and derivative mutant strains and primers used in this work are described in Supplementary Tables 1, 2, respectively. *S. aureus* strains were grown on tryptic soy agar (TSA) plates or in tryptic soy broth (TSB) at 37°C. *S. aureus* USA300 *pbp4*-null (Δpbp4), *pbp3*-null (Δpbp3), *atl*-null (Δatl), clfA-null (ΔclfA), *sasC*-null (ΔsasC), and *mreC*-null (ΔmreC) strains were constructed by allelic replacement using *Escherichia coli–S. aureus* shuttle vector pWedge, as previously described (Canfield et al., 2013). Deletion was confirmed by PCR amplification and sequencing of the chromosomal region flanking the gene of interest in USA300.

### Growth Rate Measurements

*Staphylococcus aureus* cultures were prepared by growing overnight, and then subcultured the following day. Each strain of *S. aureus* was grown in a 96-well plate at 37°C with shaking in a spectrophotometer, and growth rate was evaluated by measuring optical density at 600 nm every hour from 0 to 24 h.

### Scanning Electron Microscopy

Scanning electron microscopy (SEM) was used to characterize bacterial cell morphology and μSiM-CA bacterial propagation as previously described (Masters et al., 2019a, 2020). For cell morphology characterization, *S. aureus* cultures were grown overnight, and then subcultured and seeded onto poly-L-lysine-coated glass coverslips for 6 h before rinsing bacterial cells and fixating with 2.5% glutaraldehyde/4% paraformaldehyde in 0.1 M cacodylate buffer overnight. Similarly, μSiM-CA membranes were incubated for 6 h, as described above, and fixed with 2.5% glutaraldehyde/4% paraformaldehyde in 0.1 M cacodylate buffer overnight. Samples were postfixed in 1% osmium tetroxide, dehydrated in a graded series of ethanol to 100%, and critical point dried in a Tousimis CPD (Rockville, MD, United States). Samples were sputter coated with gold and imaged using a Zeiss Auriga Field Emission SEM (Jena, Germany) for quantification of cell diameters or qualitative assessment of bacterial propagation. ImageJ, specifically Fiji (Schindelin et al., 2012), was used to measure the maximum cell diameter across six separate SEM images per cell type, where a minimum of 20 cells were measured in each image.

### Microfluidic Silicon Membrane-Canalicular Array Propagation Experiments

Microfluidic silicon membrane-canalicular array devices were constructed as previously described (Masters et al., 2019a, 2020). Briefly, this system features a 400-nm-thick silicon nitride membrane with an array of 500-nm-sized pores fabricated by SiMPore Inc. (West Henrietta, NY, United States). High-throughput production of μSiM-CA was achieved by ALine Inc. (Rancho Dominguez, CA, United States) using laser cutting and lamination of acrylic, polyethylene terephthalate, and cyclo-olefin polymer layers bonded with pressure-sensitive adhesives, as previously described (Masters et al., 2019a). The resulting device contains defined top and bottom wells connected only through the nanoporous membrane.

The μSiM-CA device was loaded by adding 10 μl of sterile TSB to the basal chamber of the device *via* the side inlet channels, and 80 μl of pure bacterial subcultures to the apical chamber above the nanoporous membrane. *S. aureus* strains were incubated in the top chamber of the μSiM-CA at 37°C for 6 h. Following incubation in the μSiM-CA, apical (input) and basal (output) media were aspirated and outgrown overnight to expand the resultant bacterial populations and confirm or deny bacterial propagation by positive or negative culture.

### Murine Model for Implant-Associated Infection

All animal studies were performed in accordance with protocols approved by the University Committee on Animal Resources at the University of Rochester Medical Center and in accordance with the Animal Welfare Act. Surgeries were performed as previously described (Li et al., 2008; Varrone et al., 2014; Masters et al., 2020, 2021). Six-week-old, female Balb/C mice were purchased from Jackson Laboratories (Bar Harbor, ME, United States) and were acclimated for 1 week prior to surgery. Mice were housed five per cage in two-way housing on a 12-h light/dark cycle. A flat stainless steel wire with a cross section of 0.2 mm × 0.5 mm (MicroDyne Technologies, Plainville, CT, United States) was cut at 4 mm in length and bent into an L-shaped implant. Mice were anesthetized prior to surgery with xylazine (12 mg/kg) and ketamine (130 mg/kg) and were administered preoperative slow-release buprenorphine. The stainless steel pins were first sterilized, and then inoculated with an overnight culture *S. aureus* for 20 min (approximately 5.0 × 10^5^ CFU/ml). The right hind limb was shaved and washed with 70% ethanol, and then a 5-mm incision was created on the medial surface of the tibia. Next, the tibia was drilled with 30- and 26-gauge needles before carefully inserting the infected pin through the tibia. Finally, the muscle and skin were closed, and day 0 X-ray images were acquired to confirm proper pin placement (LX-60 X-Ray Cabinet, Faxitron Bioptics LLC; Tucson, AZ, United States). Mice were weighed on days 0, 3, 7, 10, and 14 postinfection to track animal health. On day 14 postinfection, mice were sacrificed, and X-ray images were obtained to evaluate postinfection septic implant loosening. Pins were determined to be stably intact within the tibia as they were originally implanted or entirely dislodged from the tibia, as previously described (Masters et al., 2020, 2021). Tibia, implant, and soft tissue were harvested and placed in sterile phosphate-buffered saline (PBS) on ice for immediate colony-forming unit (CFU) quantification and subsequent cytokine quantification or placed in neutral buffered formalin (NBF) for subsequent micro-computed tomography (μCT) imaging, followed by histology and transmission electron microscopy (TEM).

### Colony-Forming Unit Quantification

Tissue and implant CFUs were quantified as previously described (Masters et al., 2020, 2021). Infected tibia, implant, and adjacent soft tissue were harvested following animal sacrifice and placed in sterile PBS on ice. Infected tibia and soft tissue were homogenized in 3 ml of PBS in a 50-ml conical using an IKA T-10 handheld homogenizer (Wilmington, NC, United States). Implants were sonicated in 1 ml of sterile PBS for 2 min at 35 kHz (VWR Intl., Radnor, PA, United States) to dislodge adhered bacteria and then vortexed. Tissue homogenate fluid and implant sonicate fluid were serially diluted in PBS and plated on TSA. Plates were incubated overnight, and resultant colonies were counted. Infected tibia and soft tissue were weighed prior to homogenizing, and CFUs were ultimately normalized to tissue mass.

### Histologic Analysis

Histologic staining of infected and sterile tibiae was performed as previously described (Masters et al., 2020, 2021). Briefly, following fixation and μCT imaging, samples were placed in 14% EDTA for 7 days of decalcification, paraffin processed, and embedded transversely with the medial side of the tibia facing downward. Five-micrometer sections were cut and mounted on glass slides.

Slides were deparaffinized and stained with Brown–Brenn modified Gram stain to visualize gram-positive bacteria. Brown–Brenn stain results in gram-positive organisms stained dark purple, cell nuclei stained pink, and connective tissue stained yellow. Slides were digitized using a VS120 Virtual Slide Microscope (Olympus, Waltham, MA, United States). The number of staphylococcal abscess communities (SACs) were quantified and averaged across three histological levels, for four biological replicates by manually counting in Olympus OlyVIA software. The area of SACs/tibia area was quantified using a custom Analysis Protocol Package (APP) in Visiopharm (v.2019.07; Hoersholm, Denmark). The APP utilizes colorimetric histomorphometry to detect gram-positive bacteria (dark purple) to accurately quantify SAC area.

Tartrate-resistant acid phosphatase (TRAP) staining was performed to visualize TRAP^+^ osteoclasts. TRAP stain results in TRAP^+^ osteoclasts stained red/purple with a blue/green tissue background. Slides were digitized using a VS120 Virtual Slide Microscope (Olympus, Waltham, MA, United States). % TRAP area was quantified using a custom APP in Visiopharm (v.2019.07; Hoersholm, Denmark) within the whole tibia. The APP utilizes colorimetric histomorphometry to detect TRAP staining (red/purple), fast green counterstain (blue/green), and background (white) in order to accurately segment TRAP^+^ area for quantification. TRAP quantification was blinded.

### Micro-Computed Tomography Imaging and Analysis

Infected tibias were fixed in 10% NBF for 3 days at room temperature with associated soft tissue and implant left intact, then rinsed in PBS and distilled water before soft tissue was dissected, and implant was removed. Infected tibias were imaged *ex vivo* by μCT in a VivaCT 40 (Scanco Medical, Bassersdorf, Switzerland) with a 10.5-μm isotropic voxel size, using an integration time of 300 ms, energy of 55 kV, and intensity of 145 μA. Resultant DICOM files were used to create a 3D reconstruction of bone tissue using Amira software (FEI Visualization Sciences Group, Burlington, MA, United States). Bone tissue was first binarized and reconstructed by thresholding. Medial hole and lateral hole volume quantification was performed by manual segmentation of the void area and interpolating through the depth of the tibial cortex, as previously described (Masters et al., 2020, 2021).

### Transmission Electron Microscopy “Pop-Off”

Regions of interest within serially sectioned paraffin blocks of infected tibia samples, adjacent to Brown–Brenn–stained sections, were processed for TEM using the “pop-off” technique, as previously described (de Mesy Jensen and di Sant’Agnese, 1992; Masters et al., 2020, 2021). Briefly, slides were deparaffininzed in three changes xylene and then rehydrated through a graded series of ethanol to dH_2_0. Rehydrated sections were then postfixed in buffered 2.5% glutaraldehyde overnight, rinsed in distilled water, and then postfixed in 1% osmium tetroxide for 20 min at room temperature. Slides were washed, dehydrated in a graded series ethanol to 100%, infiltrated for 1 h with a 1:1 mixture of 100% ethanol and Spurr resin, and embedded overnight in 100% resin. Regions of interest were polymerized in 100% Spurr resin under an inverted BEEM capsule for 24 h at 65°C. Capsules were “popped off” slides by dipping three to four times in liquid nitrogen. Thin sections were cut at ∼70 nm and placed onto formvar carbon-coated nickel slot grids for imaging using a Hitachi 7650 TEM (Pleasanton, CA, United States). Note that original formalin fixation of bone tissue, subsequent paraffin processing and embedding, and, finally, “pop-off” for TEM resulted in suboptimal ultrastructural tissue preservation. As a result, empty canaliculi often appeared as collapsed structures, making imaging of non-infected bone tissue challenging. TEM imaging was performed for three biological replicates of all groups, Δpbp3, Δatl, ΔmreC, ΔclfA, and ΔsasC. TEM imaging was blinded to sample group assignment.

### Quantification of Local Cytokines

As mentioned, bone tissue was harvested at day 14 post-infection and homogenized on ice in 3 ml of sterile PBS. Bone homogenate was centrifuged at 13,000 rpm for 15 min at 4°C to pellet bone tissue. Supernatant was aspirated and frozen in several aliquots to reduce freeze–thaw cycles and maintain cytokine stability. The dilution factor of bone homogenate supernatant was optimized for each cytokine sandwich enzyme-linked immunosorbent assays (ELISAs). Cytokines investigated include receptor activator of nuclear factor kappa-B ligand (RANKL), interleukin-1β (IL-1β), and interleukin-6 (IL-6). Dilutions used for RANKL, IL-1β, and IL-6 were 1:5, 1:25, and no dilution, respectively. ELISA protocols were performed as per the manufacturer’s recommendations (R&D Systems, Minneapolis, MN, United States, catalog #: MTR00, MLB00C, and M6000B). Briefly, diluted bone homogenate supernatant was incubated in capture-antibody-coated wells for 2 h at room temperature. Next, wells were washed, and then incubated with secondary conjugated antibody for 2 h. Wells were washed again and incubated with substrate solution for 30 min. Finally, stop solution was added, and absorbance was read at 450 nm with wavelength correction at 570 nm. Additionally, cytokine levels were normalized to total protein measured by a Pierce Bicinchoninic Acid (BCA) Protein Assay Kit (Thermo Fisher Scientific, MA, United States) and reported as ng cytokine/mg total protein to account for variability in tibia-to-tibia size.

### Statistical Analyses

Fisher’s exact test was used for comparison of nominal data to a control group, including evaluation of implant stability. Unpaired *t*-test was used when two groups were compared, including ΔmreC vs. wild-type (WT) comparisons in Supplementary Material. Two-way analysis of variance (ANOVA) with Sidak’s post-hoc for multiple comparisons was used to compare multiple variations such as differences in growth rate. One-way ANOVA, with Dunnett’s post-hoc for multiple comparisons vs. WT was used for data such as cell sizes, CFUs, abscess quantifications, μCT analysis, % TRAP quantification, and cytokine concentrations. CFU data were log transformed to achieve normal distributions for statistical analyses. All statistics were analyzed using GraphPad Prism.

## Results

### *In vitro* Characterization of *Staphylococcus aureus* Mutants

**FIGURE 2:**
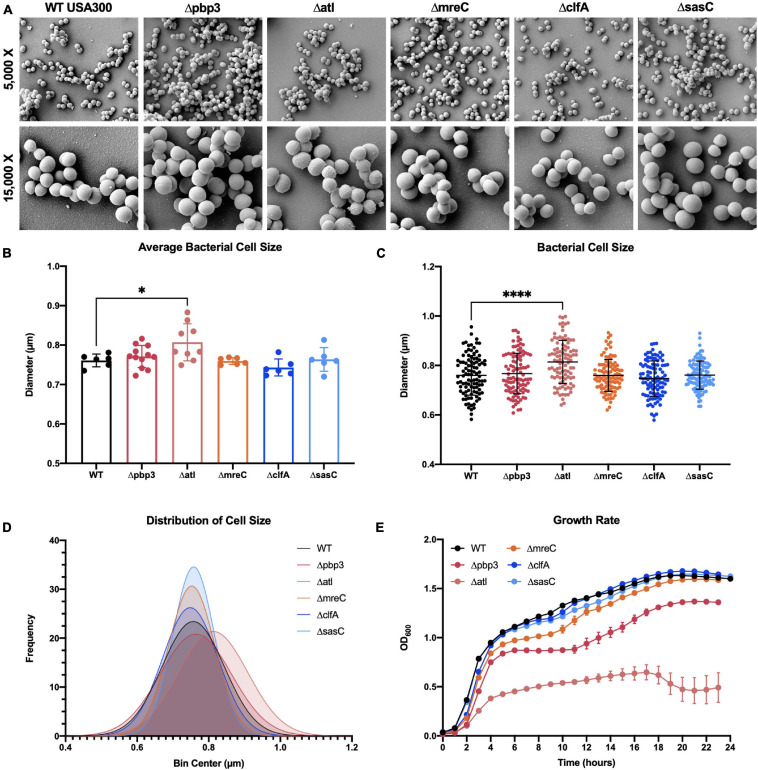
*Staphylococcus aureus* deletion mutant cell morphology and growth rate characterization. Cultures of WT USA300 and Δpbp3, Δatl, ΔmreC, ΔclfA, and ΔsasC were grown on glass coverslips and processed for scanning electron microscopy (SEM; *n* = 3 independent replicates). Representative images are shown to illustrate the absence of gross morphological differences in all mutant stains besides the Δatl mutant strain **(A)**. The Δatl mutant shows characteristic “rough” cell wall, particularly in regions of older peptidoglycan (magnified in Figure 3). Bacterial cell size was quantified as the maximum cell diameter from at least six SEM images (**B**; average size per image, *n* > 6) and the data for each bacterium with mean and SD presented (**C**; by one-way ANOVA with Dunnett’s post-hoc test for multiple comparisons vs. WT, *n* = 100). A Gaussian curve was fit to a histogram of cell sizes to visualize the distribution of cell diameters for each genotype **(D)**. WT and deletion mutants were grown in liquid culture and measured by optical density at 600 nm hourly for 24 h. The Δpbp3 mutant showed significantly reduced growth during stationary phase, and Δatl showed extremely hindered growth through all phases **(E)**. Growth rate was evaluated by *via* two-way ANOVA with Dunnett’s post-hoc for multiple comparisons vs. WT (*n* = 3, data presented as mean ± SEM). Strains Δatl and Δpbp3 were significantly different from WT at time points 3–23 and 8–23 h, respectively (*p* < 0.05).

**FIGURE 3:**
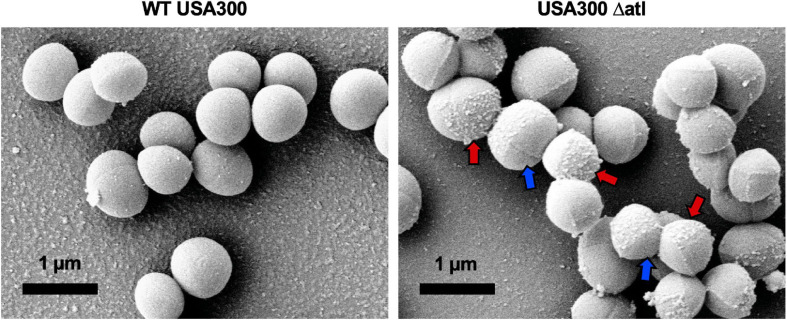
Deletion of *atl* results in aberrant *S. aureus* cell wall morphology. Cultures of WT USA300 and Δatl were grown on glass coverslips and processed for SEM imaging (*n* = 3 independent experiments). High-magnification images show the characteristic “rough” cell wall of the Δatl strain compared to the smooth cell wall of WT *S. aureus*. Red arrows denote regions of “rough” surfaces that are primarily in older regions of the cell wall, while newly separated cells show “smoother” surfaces in regions of newly synthesized cell wall, noted by blue arrows.

To expand our understanding of *S. aureus* invasion of the OLCN during osteomyelitis, we aimed to characterize the role of *S. aureus* cell wall synthesis proteins (PBP3, Atl, and MreC) and surface proteins (ClfA and SasC) *in vitro* and *in vivo*. Markerless deletion mutants were created in the methicillin-resistant strain of *S. aureus*, USA300. To eliminate potential confounding factors in downstream studies, mutant strain cell morphology and growth rate were evaluated. SEM imaging showed that all mutant strains have unchanged cell morphology (Figure 2A), with the exception of the *atl* deletion mutant (Figure 3). As the primary peptidoglycan hydrolase, loss of Atl is expected to cause an aberrant cell wall phenotype. Here, we observed *atl* deletion mutants having rougher cell wall compared to WT, which has been shown in previous work (Nega et al., 2020).

Bacterial cell size was quantified by measuring the diameters of 100 cells across at least six SEM images. Mean bacterial cell size across images (Figure 2B) and across all cells (Figures 2C,D) showed *atl* deletion resulted in ∼6% increase in bacterial cell size. Next, the *in vitro* growth rate of WT *S. aureus* and mutant strains was evaluated by optical density at 600 nm measured every hour for 24 h (Figure 2E). While a*tl* deletion resulted in an apparent decrease in growth (significantly different from WT at time points 3–23 h, *p* < 0.05), this mutant strain is known to form cell aggregates or “megaclusters” (Varrone et al., 2011, 2014) and has a tendency to fall out of suspension, thereby skewing optical density measurements (Varrone et al., 2011). Interestingly, *pbp3* deletion caused attenuated growth during stationary phase (significantly different from WT at time points 8–23 h, *p* < 0.05).

**FIGURE 4:**
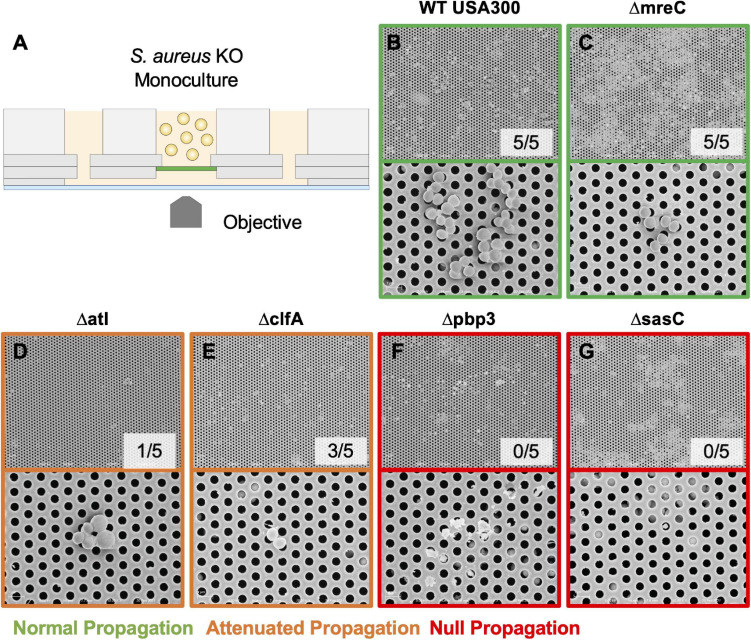
Evaluating *S. aureus* deletion mutant deformation and propagation through 0.5-μm pores in the microfluidic silicon membrane-canalicular array (μSiM-CA). Pure cultures of WT USA300, Δpbp3, Δatl, ΔmreC, ΔclfA, and ΔsasC were assayed for their ability to propagate through the 0.5-μm pores of the μSiM-CA device (*n* = 7 independent experiments) and processed for SEM imaging of the bottom surface of the membrane (*n* = 3 devices imaged per group). Experimental design is illustrated in panel **(A)**. Representative SEM images are shown at 5,000× (top panel) and 15,000× (bottom panel) for each genotype **(B–G)**, and the fraction of successful propagation to the bottom is indicated. As shown in previous work, WT bacteria readily propagated through the 0.5-μm pores following 6 h of incubation **(B)**. *S. aureus* lacking *mreC* expression showed no difference from WT **(C)**. *Atl* and *clfA* deletion resulted in “attenuated propagation,” characterized by very few bacterial cells present on the bottom surface of the membrane **(D,E)**. Finally, *pbp3* and *sasC* deletion mutants displayed “null propagation” with zero bacterial cells cultured in the bottom channel or visualized on the bottom surface of the membrane **(F,G)**. Only cellular and media debris were found on the bottom surface of membranes from Δpbp3 studies **(F)**.

The μSiM-CA *in vitro* model was used to determine the mutant strains’ ability to deform and propagate through the 0.5-μm pores of the membrane to the basal chamber of the device (Figure 4A). Note, previous work screened pools of transposon mutants in the μSiM-CA (Masters et al., 2020), while this work uses monocultures of deletion mutants. Briefly, pure cultures of WT *S. aureus* or deletion mutants were added to the apical chamber of the device, incubated for 6 h, then media within the basal chamber was outgrown to determine the presence of bacteria, and the bottom side of the membrane was imaged by SEM. Expectedly, WT *S. aureus* readily propagated through the μSiM-CA (Figure 4B). In addition, we observed that *mreC* deletion did not affect bacterial propagation, shown by positive cultures from basal media in 5/5 experiments and equivalent bacterial presence on the bottom surface of the membrane compared to WT (Figure 4C). Therefore, *mreC* is not required for OLCN invasion and included in the Supplementary Material from this point forward. However, deletion of *atl* and *clfA* showed positive cultures from basal media, with sparse bacterial presence (<10 cells) on the bottom surface of the membrane (Figures 4D,E). Most interestingly, complete loss of propagation was noted for *pbp3* and *sasC* deletion strains, with negative cultures from basal media and membranes completely devoid of bacteria (Figures 4F,G). The results of *in vitro* characterization studies for *S. aureus* cell wall synthesis and surface adhesin deletion mutants are summarized in Table 1. As mentioned, Atl is associated with both cell wall synthesis and surface adhesin functions; however, for the purpose of this work, it was organized as a cell wall synthesis protein.

**TABLE 1:** *In vitro* characterization of *S. aureus* deletion mutants across functional groups.

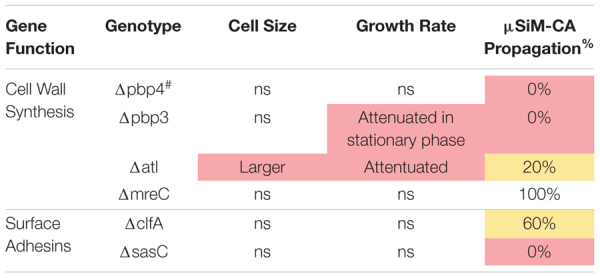

*^#^ Data adapted from, Masters et al. (2020). ^%^Percent experiments with positive cultures from basal chamber.*

### Evaluating *Staphylococcus aureus* Pathogenesis *in vivo*

**FIGURE 5:**
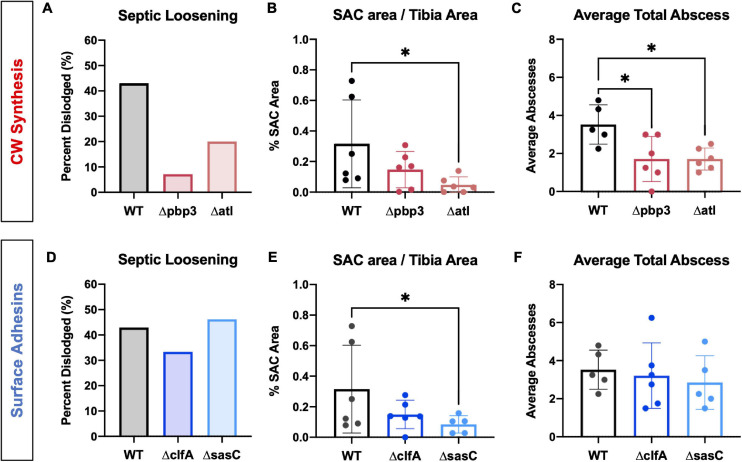
*Staphylococcus aureus* cell wall synthesis mutants show diminished septic implant loosening and significantly decreased bone marrow abscess formation, while *S. aureus* surface adhesin mutants show minimal changes in pathogenesis. L-shaped wires contaminated with WT, Δpbp3, Δatl, ΔclfA, and ΔsasC *S. aureus* were surgically implanted through the tibia of mice as previously described. X-rays were obtained at the time of sacrifice to determine if the implant remained fixed or was dislodged from the tibia as a measure of septic implant loosening, as previously described (Masters et al., 2020) (**A**,**D**; *n* = 15). While not statistically significant by Fisher’s exact test, these data show that all cell wall biosynthesis mutants demonstrate diminished septic implant loosening compared to WT (**A**, *n* = 15). Deletion of *clfA* or *sasC* did not affect septic implant loosening **(D)**. SAC area was quantified from Brown–Brenn–stained histologic sections using Visiopharm image analysis (**B**,**E**; *n* = 5–6), and average abscess number was quantified manually (**C**,**F**; evaluated by one-way ANOVA with Dunnett’s *post-hoc* test for multiple comparisons vs. WT, ^∗^ *p* < 0.05, *n* = 5–6).

Following *in vitro* characterization, WT *S. aureus* and mutant strains (Δpbp3, Δatl, ΔmreC, ΔclfA, and ΔsasC) were evaluated for pathogenesis and, ultimately, OLCN invasion *in vivo* using a murine model of implant-associated osteomyelitis (Masters et al., 2020, 2021). In this model, a stainless steel pin is left sterile or inoculated with WT *S. aureus* or mutant strains (Δpbp3, Δatl, ΔmreC, ΔclfA, and ΔsasC) before being implanted through the tibia for 14 days. CFU quantification from *in vivo* infections showed equivalent bacterial colonization in bone, soft tissue, and implants by all mutant *S. aureus* strains compared to WT (Supplementary Figure 1). Animal weight change analysis, as a measure of morbidity, also revealed no differences between the infection groups (Supplementary Figure 2). At euthanasia, examination of implant stability via X-ray imaging revealed markedly less septic implant loosening in infection by cell wall synthesis deletion mutants (Figure 5A), while surface adhesin mutant infections show similar rates of septic loosening compared to WT (Figure 5D). Deletion of *mreC* showed no changes in CFUs nor implant loosening as predicted by the μSiM-CA screen (Supplementary Figure 3).

Histopathology of infected tibiae revealed that cell wall synthesis mutants tend to form smaller (Figure 5B) and significantly less SACs (Figure 5C) within the medullary cavity of infected tibiae. On the other hand, surface adhesin mutants showed no change in total abscesses (Figure 5F), and *sasC* deletion may influence the size of SACs (Figure 5E). Again, *mreC* deletion did not change abscess formation (Supplementary Figure 3). Representative images of Brown–Brenn–stained histologic sections are summarized in Supplementary Figure 4.

**FIGURE 6:**
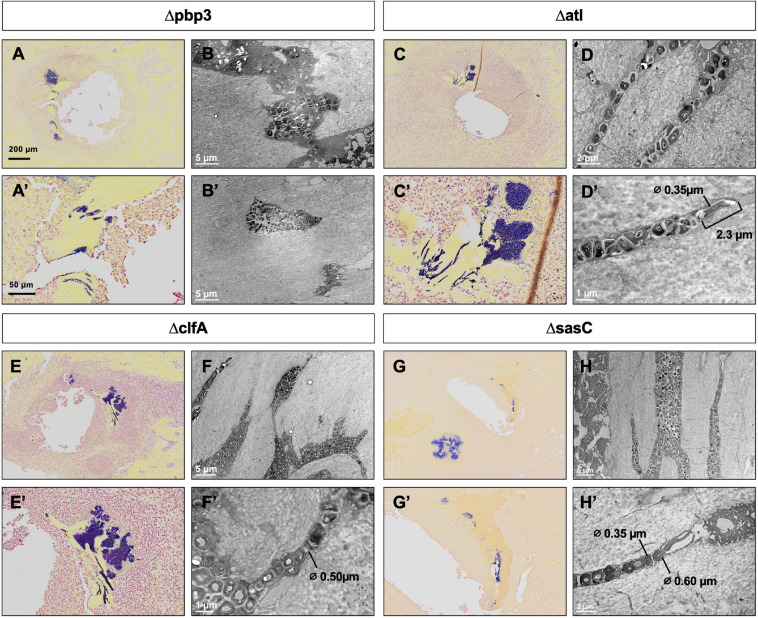
*Pbp3* deletion eliminates *S. aureus* OLCN invasion in a murine model of implant-associated osteomyelitis. Brown–Brenn–stained histology sections were used to identify necrotic bone fragments containing gram-positive bacteria (purple) in tibiae infected with Δpbp3, Δatl, ΔclfA, and ΔsasC *S. aureus*, and adjacent tissue sections (*n* = 3 tibiae per group) were used for ultrastructural analysis *via* the TEM “pop-off” method to formally interrogate OLCN invasion. Representative images of Brown–Brenn histologic sections **(A,C,E,G)** and TEM micrographs are shown for each infection genotype **(B,D,F,H)**. *Pbp3* deletion does not eliminate *S. aureus* colonization of the bone **(A,A’)**. However, Δpbp3 bacteria colonization appears to be limited to microcracks **(B)** and blood vessel canals **(B’)** but were not found in submicron canaliculi. On the other hand, deletion of *atl, clfA*, and *sasC* does not inhibit *S. aureus* invasion of canaliculi **(C–H)**. Note the submicron deformation (∅, cell diameters are measured where indicated) and linearized propagation of Δatl, ΔclfA, and ΔsasC cells within bone canaliculi **(D’,F’,H)**. Note the extreme deformation of Δatl *S. aureus* cells at the leading edge of invasion, measuring at 0.35 μm in diameter and 2.3 μm in length **(D’)**.

Previous work determined that *pbp4* deletion eliminates *S. aureus* OLCN invasion (Masters et al., 2020). Brown–Brenn–stained histologic sections were used to identify regions of *S. aureus* colonized bone for TEM “pop-off,” as previously described (Masters et al., 2020). Infected bone fragments are commonly located near the implant site (Figures 6A,C,E,G). Magnified regions show extensive colonization of the yellow-stained bone tissue by the dark-purple-stained gram-positive bacterial cells in all mutant infection groups (Figures 6A’,C’,E’,G’). While all mutants show bone colonization, as suggested by the Brown–Brenn–stained histology, blinded TEM interrogation of the infected bone revealed that only Δatl, ΔclfA, and ΔsasC strains showed evidence of bacterial colonization of the OLCN (Figures 6B,D,F,H). Samples infected with Δpbp3 *S. aureus* showed colonization of wide channels (over 2 μm, likely microcracks) and within a blood vessel lacunar space (Figure 6B’). In contrast, Δatl, ΔclfA, and ΔsasC bacteria are measured at or below ∼0.5 μm in width, within the confines of the bone canaliculi. Note the Δatl *S. aureus* cell at the leading edge of canalicular invasion deformed to 0.35 μm in width and 2.3 μm in length (Figure 6D’). As expected, *mreC* deletion did not inhibit *S. aureus* invasion of the OLCN (Supplementary Figure 3). Bacterial infection phenotype *in vivo* is summarized in Table 2.

**TABLE 2:** Evaluation of *S. aureus* deletion mutant bone infection phenotypes.

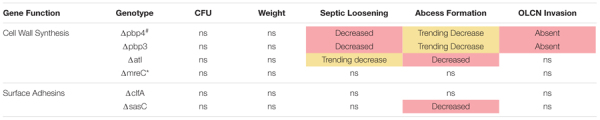

^#^Data adapted from, Masters et al. (2020). *Supplementary Data.

### Evaluating the Host Response to *Staphylococcus aureus* Bone Infection

**FIGURE 7:**
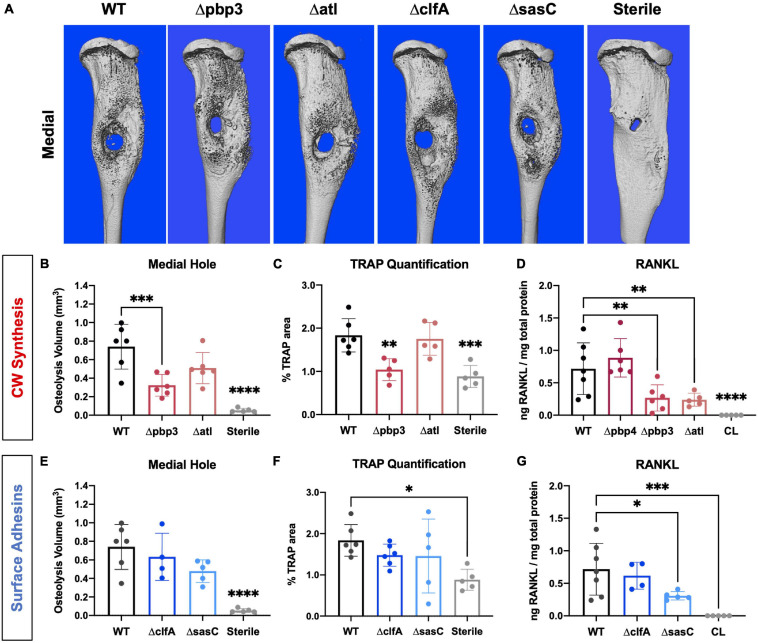
*Staphylococcus aureus* cell wall biosynthesis mutations reduce osteoclast-mediated peri-implant osteolysis compared to WT, and surface adhesin mutations do not change bone loss. Sterile or *S. aureus*-infected tibiae were harvested on day 14 postinfection for micro-computed tomography (μCT) analyses. The μCT DICOM scans were reconstructed using Amira^TM^, and medial hole volume was identified though the depth of the tibial cortex by manual segmentation and interpolated between slices. Representative 3D reconstructions of the μCT scans for all infection and sterile pin groups are shown from the medial side (**A**; *n* = 5–6). Medial hole volumes for each tibia are presented with mean ± SD for each group (**B**,**C,E**,**F**; *n* = 5–6). *Pbp3* deletion resulted in less osteolysis of the medial cortex **(B)**, similar to *pbp4* deletion shown in previous work (Masters et al., 2020). *ClfA* and *sasC* deletion did not change peri-implant osteolysis of the medial tibial cortex **(E)**. Histologic sections from sterile and infected tibiae were stained for TRAP expression to measure osteoclast prevalence. % TRAP-stained area was quantified using Visiopharm and showed a significant reduction in % TRAP staining in Δpbp4- and Δpbp3-infected tibiae and not ΔclfA and ΔsasC (**C**,**F**; *n* = 5–6 presented as mean ± SD). Local concentrations of RANKL were quantified for each infection genotype as well as within contralateral (CL) limbs of WT *S. aureus-*infected animals by measuring *via* ELISA of bone tissue homogenate **(D,G)**. Significance was evaluated by one-way ANOVA with Dunnett’s post-hoc for multiple comparisons vs. WT, ^∗^*p* < 0.05, ^∗∗^*p* < 0.01, ^∗∗∗^*p* < 0.001, ^****^p < 0.0001.

Next, the host response to infection was characterized by measuring peri-implant osteolysis and local cytokine production. First, μCT analysis was performed to quantify the extent of peri-implant osteolysis through the medial and lateral tibial cortices as previously described (Masters et al., 2020, 2021), and representative images are depicted in Figure 7A. Deletion of *pbp3* showed a significant reduction in osteolysis of the medial cortex (Figure 7B), similar to the results of *pbp4* deletion described in previous work (Masters et al., 2020). No significant changes were noted in lateral hole osteolysis with *pbp3* and *atl* deletion; however, there is distinct grouping of high and low osteolysis samples among these cohorts (Supplementary Figure 5).

Histologic staining for TRAP within tibial cross sections was performed to determine osteoclast activity within sterile and infected tibial cross sections. % TRAP area within the whole tibia was averaged across multiple histologic levels per sample. Again, *pbp3* deletion significantly reduced TRAP staining and, by extension, reduced osteoclast activity compared to WT infection (Figure 7C). Tibiae infected with Δpbp3 *S. aureus* showed statistically similar % TRAP area to sterile pin tibiae. These data support μCT quantification of peri-implant osteolysis.

To expand our understanding of peri-implant osteolysis during infection, the production of osteoclast activating cytokine, RANKL, was quantified by ELISA of infected and contralateral bone tissue homogenate at day 14 post-infection. First, contralateral tibiae showed undetectable levels of RANKL, confirming the response of a local infection versus systemic. Further, serum cytokine levels were undetectable (data not shown). Archived Δpbp4 infected tibiae were also processed for cytokine quantification. Interestingly, *pbp4* deletion showed unchanged RANKL levels compared to WT despite an apparent decrease in osteolysis and osteoclast activity, described in previous work (Masters et al., 2020). *Atl* deletion, however, showed a significant reduction in local RANKL production despite unchanged osteolysis and osteoclast presence (Figure 7D). *Pbp3* deletion resulted in a significant reduction in RANKL, as expected (Figure 7D). On the other hand, deletion of *S. aureus* surface adhesin genes showed modest changes in osteolysis and osteoclast activation. Loss of *clfA* and *sasC* did not alter medial (Figure 7E) or lateral (Supplementary Figure 5) osteolysis measured by μCT. Further, deletion of these surface adhesins did not change osteoclast presence through the tibiae (Figure 7F). Representative TRAP-stained histologic sections are summarized in Supplementary Figure 6. Interestingly, *sasC* deletion appeared to reduce RANKL expression, possibly due to hindered SAC formation (Figure 7G).

**FIGURE 8:**
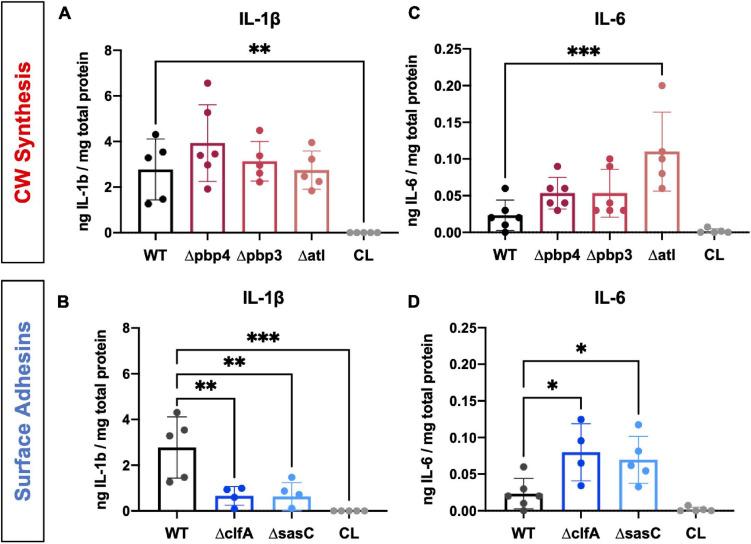
*Staphylococcus aureus* cell wall synthesis and surface adhesin mutants show significant changes in host proinflammatory cytokine production at the infection site. Local concentrations of IL-1β **(A,B)** and IL-6 **(C,D)** were quantified for each infection genotype as well as within contralateral limbs by measuring *via* ELISA of bone tissue homogenate. Significance was evaluated by one-way ANOVA with Dunnett’s post-hoc for multiple comparisons vs. WT, ^∗^*p* < 0.05, ^∗∗^*p* < 0.01, ^∗∗∗^*p* < 0.001, *n* = 4–6.

In addition to RANKL, local levels of presumed proinflammatory and osteoclast-activating cytokines, IL-1β and IL-6, were measured for WT and mutant *S. aureus* infection groups. Previous work has shown that IL-1β and IL-6 production is increased during osteomyelitis (Putnam et al., 2019). In contrast, deletion of cell wall synthesis genes, *pbp4* and *pbp3*, did not significantly alter IL-1β production despite reductions in osteolysis and osteoclast activation (Figure 8A). However, deletion of surface adhesins *clfA* and *sasC* reduced local IL-1β production (Figure 8B). Most surprisingly, deletion of *atl*, *clfA*, and *sasC* resulted in increased IL-6 production compared to WT infection (Figures 8C,D). *In vivo* host–pathogen interactions for all mutant strains are summarized in Table 3.

**TABLE 3:** Host response to infection by *S. aureus* deletion mutants.

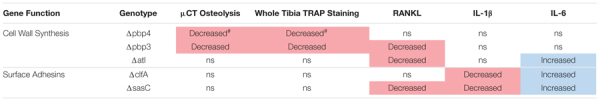

*^#^Data adapted from, Masters et al. (2020).*

## Discussion

This study investigated the role of select *S. aureus* cell wall synthesis proteins and surface adhesins in bone infection pathogenesis and invasion of the OLCN. We hypothesized that *S. aureus* cell wall synthesis machinery and surface adhesins enable durotaxis- and haptotaxis-driven bacterial cell invasion of the canalicular network in infected bone.

Previous studies identified PBP4 as a critical factor for *S. aureus* invasion of the OLCN and pathogenesis in osteomyelitis (Masters et al., 2020). PBP4 is a non-essential transpeptidase responsible for the characteristic high degree of peptidoglycan cross-linking of the *S. aureus* cell wall (Wyke et al., 1981). In this work, we evaluated PBP3, another non-essential transpeptidase of *S. aureus* (Pinho et al., 2000), for submicron deformation and OLCN invasion *in vivo*. In contrast to the results of the pooled mutant genetic screen, where *pbp3* transposon mutants showed no change in nanopore propagation, this study showed that *pbp3* deletion eliminated *S. aureus* deformation and propagation through nanopores, similar to *pbp4* deletion. Further, *in vivo* studies showed *pbp3* deletion decreased septic implant loosening and abscess formation like *pbp4*. While *pbp3* deletion did not completely eliminate colonization of bone fragments, submicron-scale invasion of canaliculi was not observed in this group, suggesting reduced OLCN invasion. Important work by Reichmann et al. (2019) revealed that PBP3 interacts with RodA, a member of the shape, elongation, division, and sporulation (SEDS) protein family, to mediate side-wall peptidoglycan synthesis in *S. aureus*. Together, this SEDS-PBP cognate pair is responsible for normal *S. aureus* elongation through the cell cycle. The observed difference in OLCN invasion with *pbp3* deletion suggests a possible role for *S. aureus* cell elongation during invasion of submicron-sized canaliculi and warrants continued studies.

Additionally, we aimed to elucidate the role of major peptidoglycan hydrolase, Atl, during *S. aureus* bone infection pathogenesis and OLCN invasion, given its opposite function to PBPs. In agreement with previous studies, *atl* deletion produced slightly larger cells with rough cell surfaces and increased cell aggregation (Wheeler et al., 2015). These factors may contribute to the observed attenuation of nanopore propagation *in vitro*. *In vivo* studies showed that *atl* deletion decreased abscess formation. Previous studies have shown that megacluster formation by *atl* deletion mutants may increase susceptibility to phagocytosis (Dalia and Weiser, 2011). Lack of phagocytosis events leads to decreased *S. aureus* dissemination through host tissues (Pollitt et al., 2018), which may hinder abscess formation in Δatl infections. While deletion of *atl* appeared to diminish nanopore propagation *in vitro* and modulate pathogenesis *in vivo*, *atl* expression was not necessary for invasion of the OLCN. Notably, *S. aureus* lacking expression of *atl* showed extensive invasion of the OLCN with extremely deformed bacterial cells measuring less than 0.5 μm in diameter and over 2.0 μm in length at the leading edge of invasion.

In order to maintain constant bacterial cell shape during growth and division, the of activity PBPs must be in balance with the activity of cell wall hydrolases to synthesize and degrade peptidoglycan during growth and division (Vermassen et al., 2019). Previous work has shown *S. aureus* lacking *pbp4* expression has less cross-linked peptidoglycan and, in consequence, decreased cell wall stiffness (Loskill et al., 2014). Consistent with these findings, *atl* deletion results in increased peptidoglycan cross-linking and increased cell wall stiffness (Wheeler et al., 2015). Together, this work suggests that sufficient peptidoglycan cross-linking and cell wall rigidity are necessary for *S. aureus* invasion of bone canaliculi *in vivo*, where *pbp4* deletion and decreased cross-linking eliminate OLCN invasion and *atl* deletion and increased cross-linking allows OLCN invasion with notably elongated cells.

In continuation, we hypothesized that the transmembrane protein MreC could be involved in *S. aureus* deformation from cocci to rod shape during OLCN invasion. In rod-shaped bacteria, actin homolog MreB polymerizes to form cytoskeletal filaments that localize with MreC and PBPs to coordinate peptidoglycan synthesis and cell wall elongation (Kruse et al., 2005; Carballido-López and Formstone, 2007). Although MreB is not present in coccoid cells, MreC has been conserved in *S. aureus* without a known function (Tavares et al., 2015). Our previous work showed that a *mreC* transposon insertion mutant trended toward decreased ability to propagate through nanopores (Masters et al., 2020). However, reevaluation of *mreC* deletion in this work with a monoculture μSiM-CA experiment showed no change in nanopore propagation, suggesting a possible false-positive identification of *mreC* in the previous study. Further confirming these results, the *mreC* deletion mutant showed extensive invasion of the OLCN *in vivo*, thereby concluding that *mreC* expression is not a critical factor for *S. aureus* deep bone invasion.

Lastly, surface adhesins, ClfA and SasC, were investigated as potential modulators of *S. aureus* haptotaxis in bone infection and OLCN invasion. ClfA was selected because it showed a modest decrease in μSiM-CA nanopore propagation in previous work and is important in bone infection pathogenesis in obese/type 2 diabetic mice (Farnsworth et al., 2017). Further, ClfA is an important virulence factor that has been the target of antibody-based *S. aureus* vaccine therapies, showing promising results in biofilm prevention *in vitro* and preventing disease progression in animal models of endocarditis and sepsis (Domanski et al., 2005; Tkaczyk et al., 2016). In addition, previous work identified SasC as a necessary gene for nanopore propagation *in vitro* (Masters et al., 2020). SasC is known to promote bacterial attachment, cell–cell aggregation, and biofilm formation (Schroeder et al., 2009; Zhu et al., 2020). We hypothesized that these surface proteins contribute to the initial *S. aureus* attachment and invasion of cortical bone during the establishment of osteomyelitis. Despite a suggested role of nanopore propagation *in vitro*, *in vivo* studies demonstrated that expression of *clfA* or *sasC* is not necessary for *S. aureus* invasion and colonization of the OLCN. While this finding does not support our initial hypothesis for the role of surface adhesins in OLCN invasion by haptotaxis, it is possible that the deletion of a single surface adhesin may not be sufficient for the observation of a significant phenotype *in vivo*. Given the variety of surface adhesins expressed by *S. aureus* (Foster and Höök, 1998; Foster et al., 2014), their functions are often redundant. Further, like most *in vitro* models, the μSiM-CA does not fully mimic the *in vivo* physiological properties of bone and specifically lacks ECM ligands for cell adherence. Therefore, the *in vitro* model may not accurately predict haptotaxis differences as expected *in vivo*.

Next, we measured pathogenic bone loss by quantifying osteolytic bone volume, relative osteoclast presence, and production of osteoclast-stimulating cytokine, RANKL. Chronic inflammation due to osteomyelitis can cause a sustained release of proinflammatory cytokines from innate and adaptive immune cells, resulting in osteoclast activation and pathogenic bone loss (Amarasekara et al., 2015). Generally, deletion of cell wall synthesis genes influenced pathogenic bone loss more so than the deletion of surface adhesins. Specifically, we showed that *pbp4* and *pbp3* deletion both modulate the volume of peri-implant bone loss and osteoclast activity within infected tibiae. Initially, we hypothesized that *S. aureus* bone invasion triggered osteocyte death and ensuing RANKL production resulting in osteoclast-mediated bone loss (Andreev et al., 2020). Contrary to our hypothesis, deletion of *pbp4* showed no differences in RANKL production, despite the loss of OLCN invasion, while *atl* deletion significantly reduced RANKL production with no effect on OLCN invasion, suggesting that changes in bone osteolysis cannot be explained by RANKL production alone.

To further interrogate these dichotomous observations, we measured the production of proinflammatory cytokines, IL-1β and IL-6. Putnam et al. (2019) showed that local production of IL-1β and IL-6 is elevated at multiple time points throughout a 14-day infection in a murine model of posttraumatic *S. aureus* osteomyelitis. Further, IL-1β and IL-6 are known to accelerate osteoclastogenesis and bone resorption (Amarasekara et al., 2018). Interestingly, loss of PBP4 and PBP3 did not change IL-1β production, while decreased bone loss, abscess formation, and OLCN invasion were observed. In contrast, loss of surface adhesins, ClfA and SasC, demonstrated significantly decreased IL-1β production despite not influencing bone loss and OLCN invasion. ClfA is known to be an immunogenic protein capable of stimulating a robust antibody response (Nishitani et al., 2015); therefore, loss of ClfA may influence host recognition of the pathogen and decreased cytokine production. While the immunogenicity of SasC has not been tested, these data suggest this surface protein may also influence host–pathogen interactions.

Surprisingly, IL-6 was elevated in infections by *clfA, sasC*, and *atl* deletion mutants. IL-6 is often grouped with IL-1β and RANKL as a proinflammatory and osteoclast-activating cytokine (Steeve et al., 2004); however, it is a pleiotropic cytokine with a variety of functions in host responses (Kishimoto, 1989). In fact, IL-6 may act as an anti-inflammatory mediator during acute inflammation by suppressing production of other proinflammatory cytokines (Xing et al., 1998) and initiating neutrophilic resolution (Kaplanski et al., 2003; Gabay, 2006). Again, ClfA and Atl are known to be immunogenic in experimental and clinical sera (Gedbjerg et al., 2013; Nishitani et al., 2015; Kates et al., 2020) and exhibit immunomodulatory characteristics during infection. Our results may suggest an anti-inflammatory role of IL-6 in late-stage *S. aureus* bone infection, and its production may be controlled by host recognition of *S. aureus* surface proteins. Taken together, characterization of pathogenic bone loss reinforces the importance of cell wall synthesis machinery in osteomyelitis pathogenesis. However, our results show the host response to infection is heterogeneous and multifaceted. A major limitation of our cytokine analysis is that we only looked at one time point postinfection (day 14). As cytokine levels are known to be dynamically regulated over the course of infection, no conclusions on the functional significance of our single-time point studies can be made, and further in-depth studies are warranted to investigate these host responses during the entire course of *S. aureus* osteomyelitis.

To conclude, this study showed that cell wall synthesis machinery can modulate *S. aureus* pathogenesis in osteomyelitis. This is supported by significant changes in OLCN invasion, abscess formation, and pathogenic bone loss with loss of PBP4, PBP3, or Atl. We postulate that *S. aureus* cell wall composition and peptidoglycan homeostasis are key factors for submicron invasion of canaliculi. Further, *S. aureus* proteins responsible for adhesin to bone and/or ECM molecules are known to be redundant, and therefore, it is unlikely that the role of haptotaxis can be captured in single gene deletion studies.

## Data Availability Statement

The original contributions presented in the study are included in the article/Supplementary Material, further inquiries can be directed to the corresponding author.

## Ethics Statement

The animal study was reviewed and approved by the University Committee on Animal Resources at the University of Rochester Medical Center.

## Author Contributions

EM, GM, and ES conceived the study. EM performed all the experiments and wrote the manuscript. ALG assisted with creation of deletion mutants and experimentation. LH assisted with the experimentation. KDB and CG performed the electron microscopy. JM, HA, SG, and ES supervised the study. All authors contributed to the final version.

## Conflict of Interest

JM is a founder of SiMPore, an early-stage company commercializing ultrathin silicon-based technologies. The remaining authors declare that the research was conducted in the absence of any commercial or financial relationships that could be construed as a potential conflict of interest.

## Publisher’s Note

All claims expressed in this article are solely those of the authors and do not necessarily represent those of their affiliated organizations, or those of the publisher, the editors and the reviewers. Any product that may be evaluated in this article, or claim that may be made by its manufacturer, is not guaranteed or endorsed by the publisher.
