# Supplementary material for: Staphylococcus aureus Cell Wall Biosynthesis Modulates Bone Invasion and Osteomyelitis Pathogenesis
Source: Front Microbiol. 2021 Aug 16;12:723498. doi: 10.3389/fmicb.2021.723498 (PMC8415456; doi:10.3389/fmicb.2021.723498)
Supplement: Supplementary file 1 [file Data_Sheet_1.PDF]

Supplemental Data

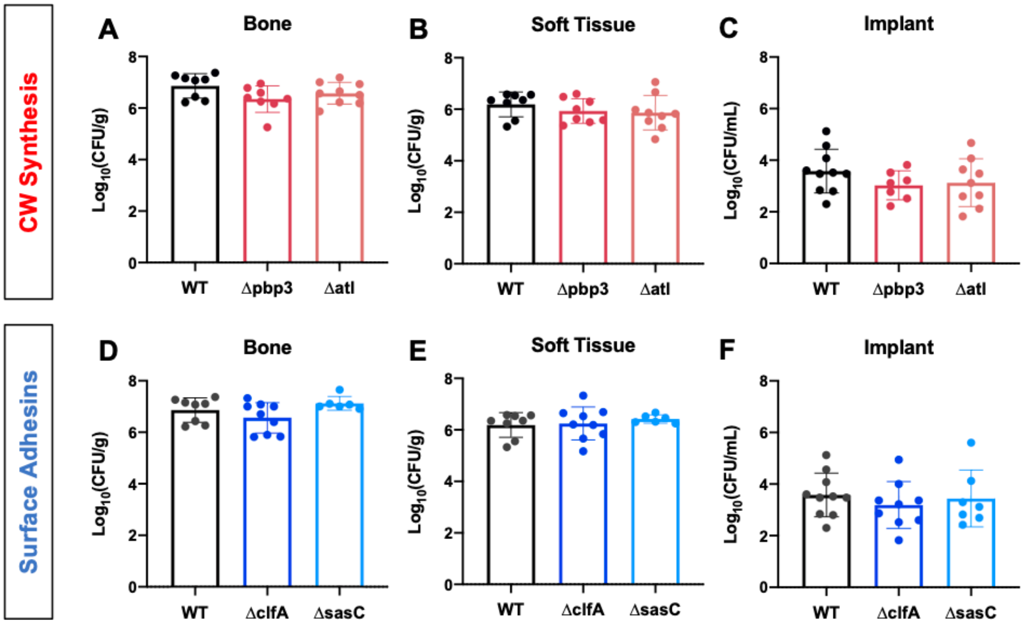

Figure S1. All *S. aureus* mutants colonize implants, bone and soft tissue with similar bacterial loads to WT.

L-shaped wires contaminated with WT, Δpbp3, Δatl, ΔclfA and ΔsasC *S. aureus* were surgically implanted through the tibia of mice as previously described. Following sacrifice, tibiae were harvested and CFUs were quantified from homogenized bone and soft tissue and from sonicated implants. CFU data for each mouse is shown with the mean +/- SD (A-F). No significant differences in CFUs between any groups were found on the implant or in bone and soft tissue (evaluated by one-way ANOVA, with Tukey's post-hoc for multiple comparisons).

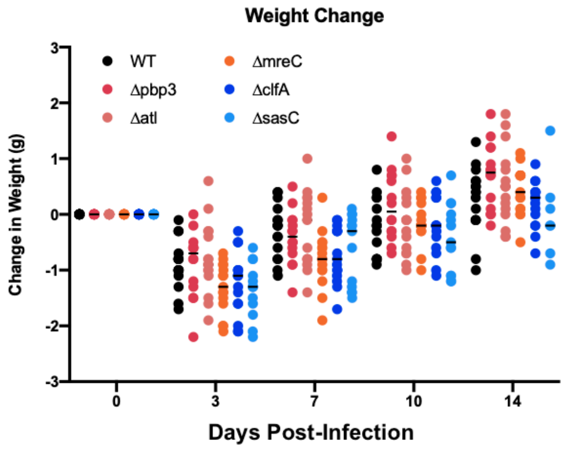

Figure S2. Animal weight was equivalent for all infection groups.

Following initial implantation of contaminated L-shaped wires (day 0), animal weight was measured on days 3, 7, 10 and 14 post-infection for WT and mutant infection groups. Change in weight from day 0 is plotted for each animal. No significant differences in weight change were found (evaluated by two-way ANOVA, with Dunnett's post-hoc for multiple comparisons vs WT).

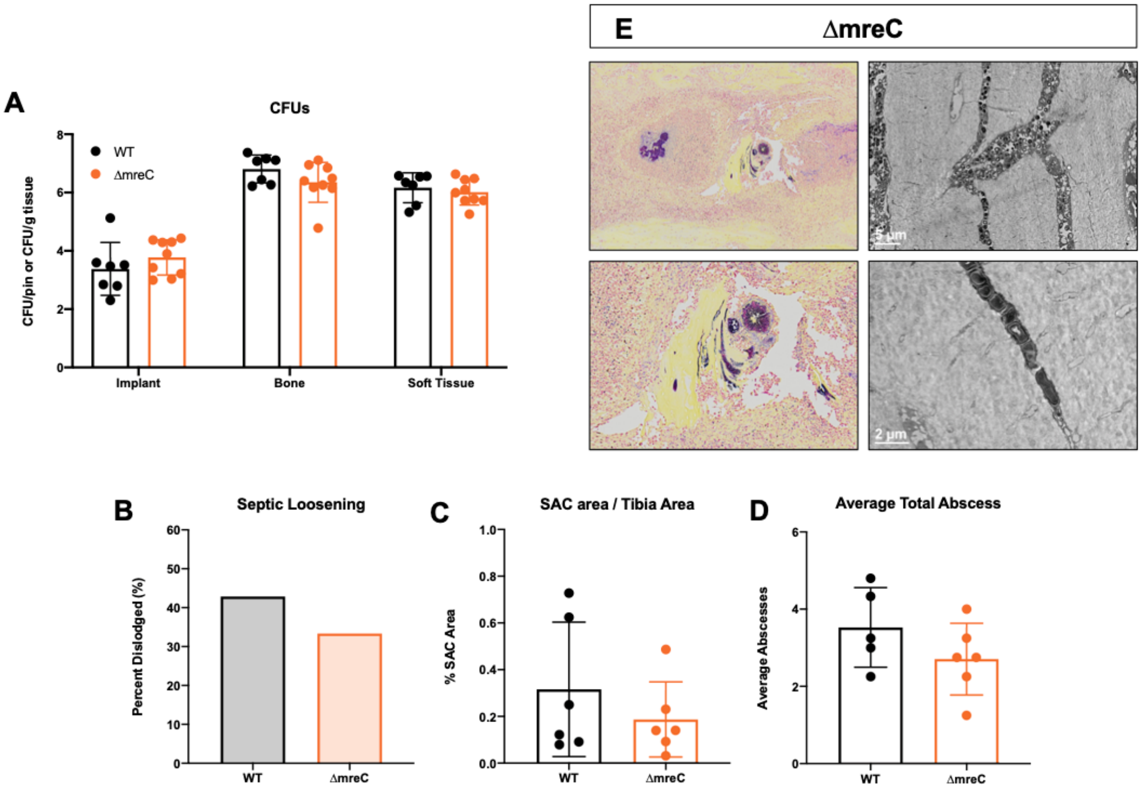

Figure S3. *MreC* deletion does not change bacterial burden, abscess formation and OLCN invasion.

CFUs were quantified from WT and ΔmreC infected implants, bone and soft tissue as described (A). No changes in implant loosening were observed by X-ray imaging at 14 days post-infection (B). Infected tibiae were also processed for histologic sectioning and staining. Brown-Brenn staining for Gram-positive bacteria was used to quantified % SAC area via Visiopharm image processing (C) and Average total abscesses were manually counted across multiple levels per sample (D). Subsequent TEM pop-off of ΔmreC infected bone confirmed bacterial invasion of the OLCN in vivo (E). Note the sub-micron scale deformation of *S. aureus* within a canaliculus (bottom right TEM). Significance evaluated by unpaired T-test.

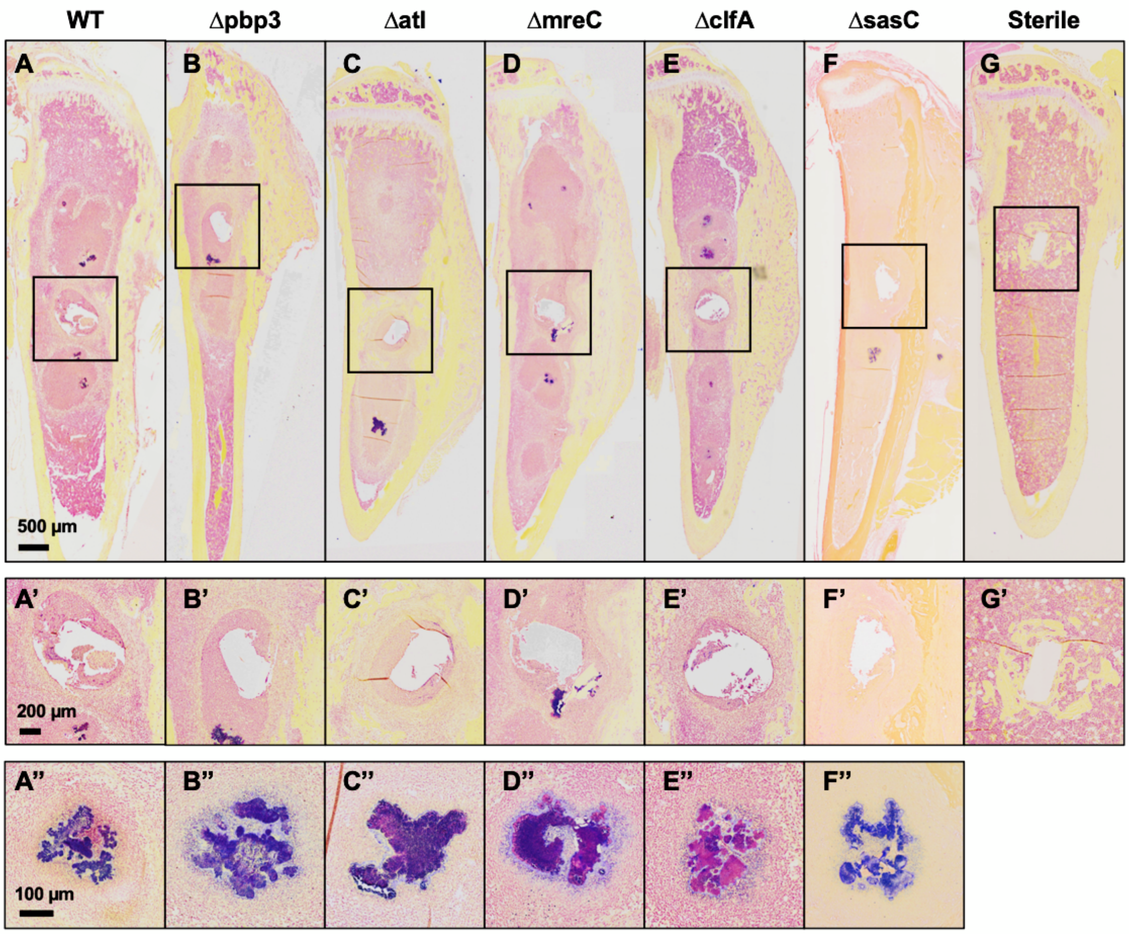

Figure S4. Histologic identification of Gram-positive bacteria at the implant site and through the bone marrow cavity.

Infected tibiae were processed for Brown-Brenn histologic staining, and representative tissue sections from each group are shown at 1x (A-G). Boxed regions are magnified at 4x to show the peri-implant reaction (A'-G') and representative abscesses from each group are shown at 10x (A''-F''). Note the dark purple-stained Staphylococcal abscess communities (SACs) and the extensive granulation tissue around the implant site in each infection group. In contrast, sterile implant tibiae lack abscesses and show evidence of osseous integration around the implant with new woven bone formation (yellow bone) (G, G'). Histologic analyses showed *pbp3* and *atl* deletion results in significantly less abscess formation.

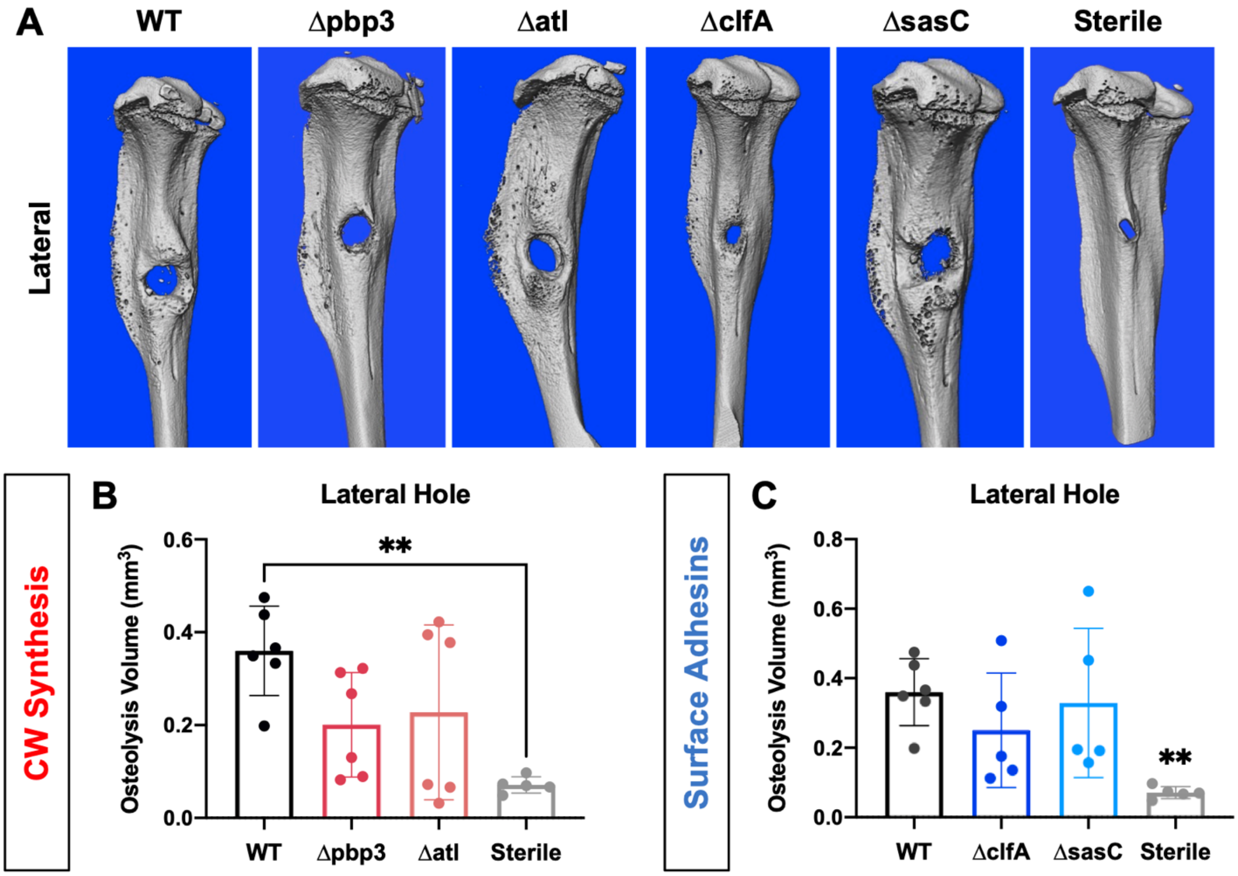

Figure S5. *S. aureus* cell wall biosynthesis and surface adhesin mutations do not significantly alter peri-implant osteolysis of the lateral tibial cortex.

Sterile or *S. aureus* infected tibiae were harvested on day 14 post-infection for μCT analyses as previously described. Representative 3D reconstructions of the μCT scans for all experimental groups are shown from the lateral side (A; n = 5-6). Lateral hole volumes for each tibia are presented with mean +/- SD for each group (B, C; n = 5-6). While no significant differences were observed in osteolysis of the lateral cortex, note the grouping of high and low lateral hole volumes in Δpbp3 and particularly in Δatl infection groups (B). *ClfA* and *sasC* deletion did not change peri-implant osteolysis of the lateral tibial cortex (C). Significance was evaluated by one-way ANOVA with Dunnett's post-hoc for multiple comparisons vs WT, *$p < 0.05$, **$p < 0.01$, ***$p < 0.001$, ****$p<0.0001$.

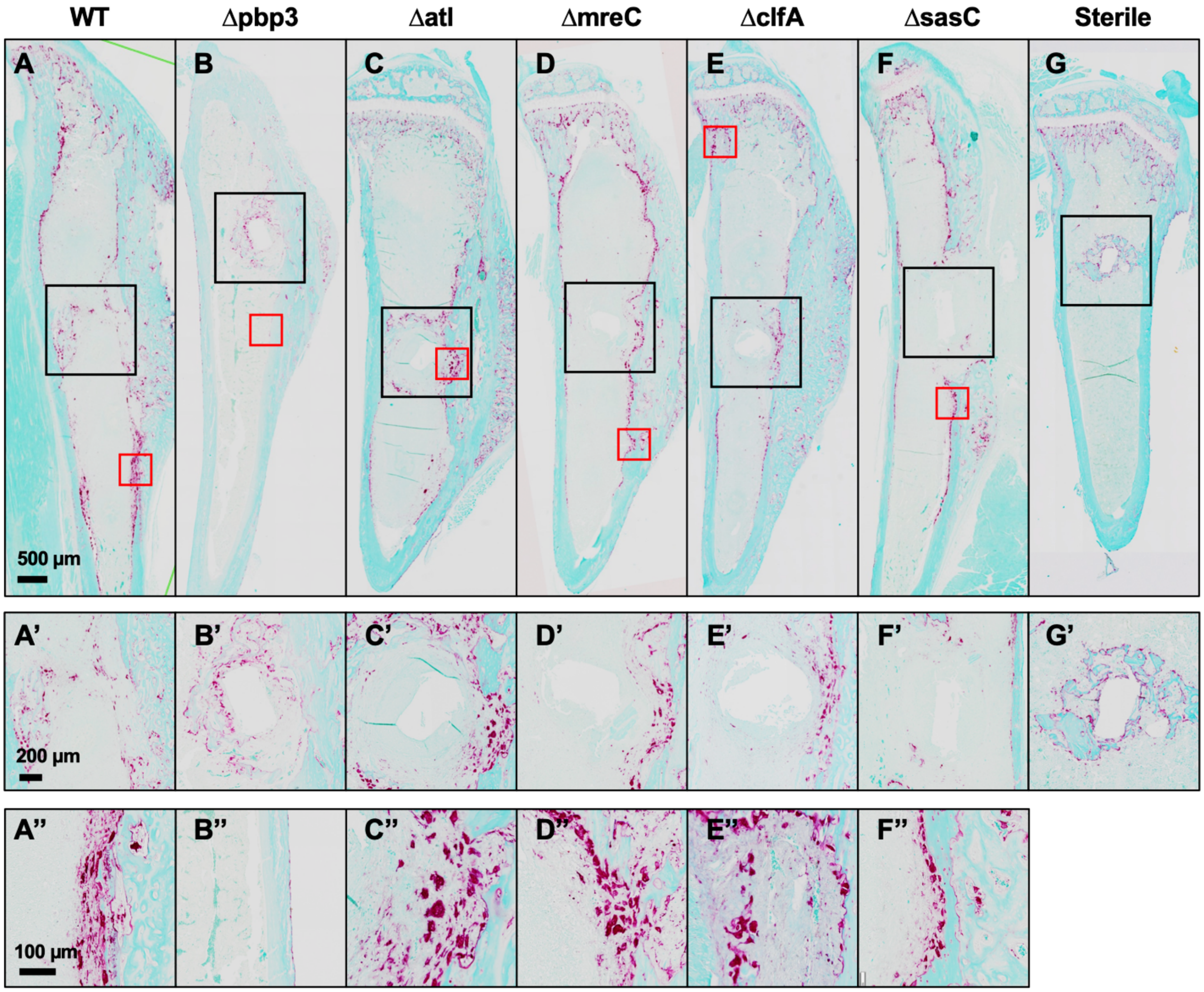

Figure S6. Histologic staining for TRAP$^+$ osteoclasts.

Infected tibiae were processed for TRAP histologic staining, and representative tissue sections from each group are shown at 1x (A-G). Boxed regions are magnified at 4x to show the peri-implant reaction (A'-G') and representative TRAP+ osteoclasts on bone surfaces from each group are shown at 10x (A''-F''). Note the absence of large, activated osteoclasts in Δpbp3 infected tibiae and in sterile tibiae (B, B'', G). Further, newly woven bone surrounding the sterile implant site shows TRAP+ staining due to dynamic bone remodeling (G').

Table S1. Strains used in this work.

USA300 Strains	Description	Reference
USA300	*S. aureus* Methicillin Resistant Isolate	(1)
USA300 Δpbp3	Δpbp3 deletion mutant of USA300	This Study
USA300 Δatl	Δatl deletion mutant of USA300	(2)
USA300 ΔclfA	ΔclfA deletion mutant of USA300	(3)
USA300 ΔsasC	ΔsasC deletion mutant of USA300	This Study
USA300 ΔmreC	ΔmreC deletion mutant of USA300	This Study
USA300 Δpbp4	Δpbp4 deletion mutant of USA300	(4)

Table S2. Primers used in this work.

Allelic Exchange Primers	Sequence	
pbp3UpF_X	TTCTGGGAGTTACTTTCACC	This work
pbp3UpR_X	CTCTTGTTAATTGTATTTTGAACG	This work
pbp3DnF_X	CAAATCAGCCTGTACCACC	This work
pbp3DnR_X	TGTTGTCATGATGAATTATGCG	This work
clfAUpF_X	AATCTAGGATATCAACGCGC	This work
clfAUpR_X	TGCATCTGCTTCTTTACTGC	This work
clfADnF_X	ACAGGTTCTGAAGATGAAGC	This work
clfADnR_X	TTCAAAGCCTCATCTTCAGC	This work
mreCUpF_X	ACTTCTAATAATAAGGTGCTGGC	This work
mreCUpR_X	AGTCGTTACAAGTGGATTAGC	This work
mreCDnF_X	CACTGATCCGGATTTCTTGC	This work
mreCDnR_X	AAAGGCCCAGAACTTATTCG	This work
sasCUpF_apaI	TGGTATCAGTGGTCCAGCTG	This work
sasCUpR_sacII	TGATTACAGGTGCGGCTTTG	This work
sasCDnF_sacII	TCGCTTTGTACATTATTATCCG	This work
sasCDnR_eagI	GATGAAGAAACAGCAATGGG	This work

Primers for Verification and Sequencing	Sequence	
pbp3_outF	CATGACAGACATCATAACACG	This work
pbp3_outR	TTTCTTAACCATGTGTCTGC	This work
clfA_outF	CAAGCATTAGAACGAGAAGC	This work
clfA_outR	TTCATATCTTTCATGAGATAATCC	This work
mreC_outF	ATTAAGGCTGAAGACTGAGG	This work
mreC_outR	TAGTGTATGGTTGACGATGG	This work
sasC_outF	CAAGTGCACTCGTAACAGG	This work
sasC_outR	GAGGGAGCGTATATTTTTAGC	This work

Supplemental References

1. Diep BA, Gill SR, Chang RF, Phan TH, Chen JH, Davidson MG, Lin F, Lin J, Carleton HA, Mongodin EF. 2006. Complete genome sequence of USA300, an epidemic clone of community-acquired meticillin-resistant Staphylococcus aureus. The Lancet 367:731-739.
2. Varrone JJ, de Mesy Bentley KL, Bello-Irizarry SN, Nishitani K, Mack S, Hunter JG, Kates SL, Daiss JL, Schwarz EM. 2014. Passive immunization with anti-glucosaminidase monoclonal antibodies protects mice from implant-associated osteomyelitis by mediating opsonophagocytosis of Staphylococcus aureus megaclusters. Journal of Orthopaedic Research 32:1389-1396.

3. Farnsworth CW, Schott EM, Jensen SE, Zukoski J, Benvie AM, Refaai MA, Kates SL, Schwarz EM, Zuscik MJ, Gill SR. 2017. Adaptive upregulation of Clumping Factor A (ClfA) by S. aureus in the obese, type 2 diabetic host mediates increased virulence. Infection and immunity:IAI. 01005-16.
4. Masters, E.A., de Mesy Bentley, K.L., Gill, A.L., Hao, S.P., Galloway, C.A., Salminen, A.T., Guy, D.R., McGrath, J.L., Awad, H.A., Gill, S.R. and Schwarz, E.M., 2020. Identification of Penicillin Binding Protein 4 (PBP4) as a critical factor for Staphylococcus aureus bone invasion during osteomyelitis in mice. PLoS Pathogens, 16(10), p.e1008988.
